# Spatio-temporal impacts of aerial adulticide applications on populations of West Nile virus vector mosquitoes

**DOI:** 10.1186/s13071-021-04616-6

**Published:** 2021-02-24

**Authors:** Karen M. Holcomb, Robert C. Reiner, Christopher M. Barker

**Affiliations:** 1 Department of Pathology, Microbiology, and Immunology, School of Veterinary Medicine, University of California, Davis, CA 95616 USA; 2 Institute for Health Metrics and Evaluation, University of Washington, Seattle, WA 98121 USA

**Keywords:** Adulticide, Aerial spraying, *Culex tarsalis*, *Culex pipiens*, GAM, Generalized additive models, Spatial-temporal model, Mosquitoes, Mosquito-borne disease, West Nile virus

## Abstract

**Background:**

Aerial applications of insecticides that target adult mosquitoes are widely used to reduce transmission of West Nile virus to humans during periods of epidemic risk. However, estimates of the reduction in abundance following these treatments typically focus on single events, rely on pre-defined, untreated control sites and can vary widely due to stochastic variation in population dynamics and trapping success unrelated to the treatment.

**Methods:**

To overcome these limitations, we developed generalized additive models fitted to mosquito surveillance data collected from CO_2_-baited traps in Sacramento and Yolo counties, California from 2006 to 2017. The models accounted for the expected spatial and temporal trends in the abundance of adult female *Culex * (*Cx.*)* tarsalis* and *Cx. pipiens* in the absence of aerial spraying. Estimates for the magnitude of deviation from baseline abundance following aerial spray events were obtained from the models.

**Results:**

At 1-week post-treatment with full spatial coverage of the trapping area by pyrethroid or pyrethrin products, *Cx. pipiens* abundance was reduced by a mean of 52.4% (95% confidence intrval [CI] − 65.6, − 36.5%) while the use of at least one organophosphate pesticide resulted in a mean reduction of 76.2% (95% CI − 82.8, − 67.9%). For *Cx. tarsalis*, at 1-week post-treatment with full coverage there was a reduction in abundance of 30.7% (95% CI − 54.5, 2.5%). Pesticide class was not a significant factor contributing to the reduction. In comparison, repetition of spraying over three to four consecutive weeks resulted in similar estimates for *Cx. pipiens* and estimates of somewhat smaller magnitude for *Cx. tarsalis.*

**Conclusions:**

Aerial adulticides are effective for achieving a rapid short-term reduction of the abundance of the primary West Nile virus vectors, *Cx. tarsalis* and *Cx. pipiens*. A larger magnitude of reduction was estimated in *Cx. pipiens*, possibly due to the species’ focal distribution. Effects of aerial sprays on *Cx. tarsalis* populations are likely modulated by the species’ large dispersal ability, population sizes and vast productive larval habitat present in the study area. Our modeling approach provides a new way to estimate effects of public health pesticides on vector populations using routinely collected observational data and accounting for spatio-temporal trends and contextual factors like weather and habitat. This approach does not require pre-selected control sites and expands upon past studies that have focused on the effects of individual aerial treatment events.
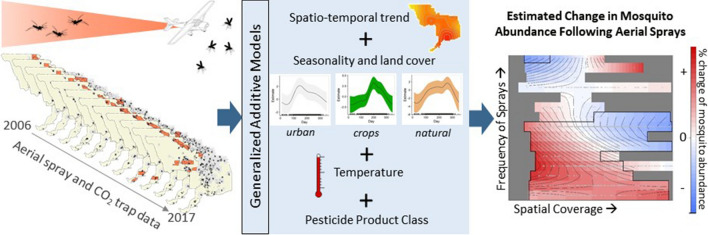

## Background

West Nile virus (WNV; genus *Flavivirus*, family *Flaviviridae*) causes a potentially fatal, neuroinvasive mosquito-borne disease [1]. It is maintained in an enzootic cycle between birds and mosquitoes [2, 3], predominantly in the genus *Culex* [4], and can spill over to infect horses and humans, both of which are dead-end hosts vulnerable to disease [5]. *Culex* (*Cx.*)* tarsalis* and *Cx. pipiens* complex mosquitoes are the primary enzootic and epizootic vectors in California [6, 7]. While 80% of human infections are asymptomatic, clinical manifestations can include acute febrile illness, encephalitis, flaccid paralysis and death [8]. Often the severe form results in long-term physical and mental disabilities [9]. WNV invaded California in 2003 and has become endemic [7]. An average of 238 neuroinvasive cases occur statewide annually, with approximately one-third occurring in the Central Valley, where the landscape is dominated by large-scale agriculture punctuated by cities and small towns [10].

As no human vaccine exists against WNV, prevention of human diseases relies primarily on personal protective measures (i.e. wearing long sleeves, using insect repellent and avoiding the dawn/dust periods when mosquitoes bite) and vector control by local vector control districts or health departments [11, 12]. In periods of epidemic risk when large numbers of WNV-infected *Culex* mosquitoes are detected near human population centers, large-scale aerial applications of insecticides are utilized to rapidly reduce the abundance of adult mosquitoes and disrupt virus transmission cycles, thereby reducing zoonotic transmission risk [13].

Three main classes of pesticide products have been licensed for use in aerial spray applications in California: pyrethrins, pyrethroids and organophosphates [12, 14, 15]. Pyrethrins are naturally derived insecticides from chrysanthemum flowers (*Chrysanthemum cineriaefolium*) that inactivate sodium channels in the insect nervous system, resulting in paralysis and death [15]. Pyrethroids are synthetically derived pyrethrins with a similar mode of action and longer half-life. Organophosphates inhibit acetylcholinesterase, affecting neurotransmission and causing uncontrolled nerve activation and death in insects [14].

A standard method for evaluating the efficacy of an aerial spray event compares pre- to post-treatment mosquito trap counts inside the treatment zone* versus* changes for the same period in an adjacent unsprayed control area [16]. This method, first proposed by Mulla et al. [17], has been adapted to and widely used in evaluating the efficacy of aerial spraying for reducing the abundance of female mosquitoes and has been extended to assess changes in other indicators of risk, namely infection prevalence in mosquitoes, human cases and reported dead birds, with WNV infection [16]. However, reported estimates vary widely, with some studies even indicating occasional increases in trap counts following spray events [18–21].

Despite its wide use, the assumptions behind Mulla’s formula are often violated, resulting in confounded estimates. First, treatment and control sites are often not independent due to the spatial connectivity of populations with mosquito dispersal and immigration [22]. With the connectivity and potential drift of pesticides* via* wind, there is the potential that insecticide sprays have wider population impacts than just the targeted spray zone [20]. Second, the difference in pre- to post-trap count ratios in and between areas are not solely due to control measures, but rather are impacted by weather, seasonality in mosquito populations, differential presence of larval breeding sources or simply stochastic variation in trapping success [18, 20, 21]. Overall, Mulla’s formula neglects both the spatio-temporal structure of mosquito populations and the external factors impacting the random volatility of mosquito trapping success.

To overcome the limitations of assessing the efficacy of aerial sprays on the individual spray event basis, we paired long-term surveillance and vector control records (12 years) from Sacramento-Yolo Mosquito and Vector Control District (SYMVCD) in California to capture baseline spatio-temporal mosquito population dynamics and estimate the magnitude and duration of the impacts of aerial sprays on the abundance of *Cx. pipiens* and *Cx. tarsalis*, the predominant WNV vectors in California. We chose a generalized additive modeling (GAM) framework to capture the nonlinear population dynamics and associations inherent to mosquito collections.

## Methods

### Study area

**Fig. 1:**
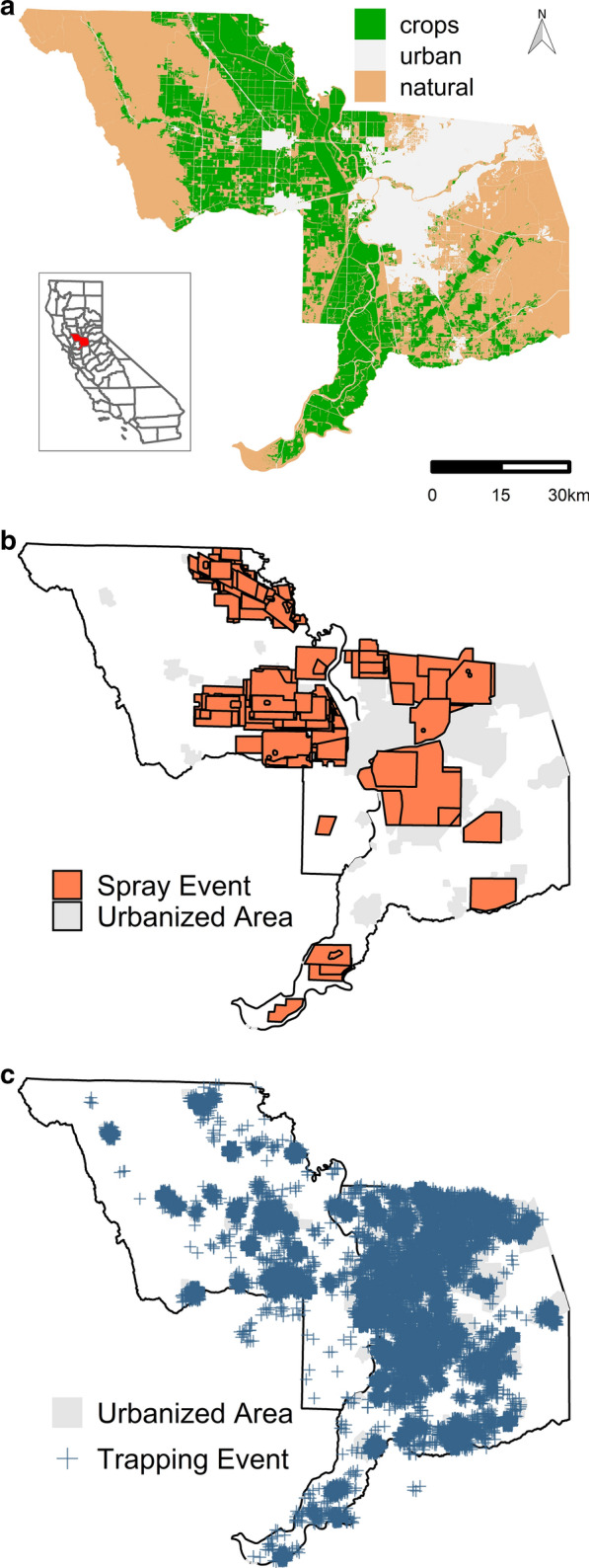
Land cover, aerial sprays and mosquito collections (2006–2017) in Sacramento and Yolo counties, California. **a** Distribution of cultivated crops (primarily rice), urban, and other natural land cover types across the study area. Land cover categories were derived from the 2011 National Land Cover database [34]. Inset highlights the location of these counties in the state of California.** b**,** c** Location of zones targeted for aerial treatment applications (**b**) and of CO_2_-baited mosquito traps (**c**) during 2006–2017 in Sacramento and Yolo counties. Each polygon (**b**) and point (**c**) represents a single spray or trapping event, respectively. A random shift of ≤ 1 km was applied to trap locations for visualization of repeated trapping at the same location across time

The study area encompasses Sacramento and Yolo counties, California (Fig. 1) which have a combined area of approximately 5,126 km^2^ and a population of approximately 1.73 million people in 2016 [23]. Sacramento County is 34.12% urban and 65.88% rural, with the majority of urban areas consisting of the concentrated Sacramento urban center and surrounding suburbs. In comparison, Yolo County is 4.61% urban and 95.39% rural, with smaller, more dispersed urban areas [24]. These counties, located in the northern part of California’s Central Valley, are characterized by a Mediterranean climate, with hot, dry summers (July mean temperature: 25.8 °C, May-September mean total rainfall: 3.18 cm) and mild, rainy winters (January mean temperature: 9.6 °C, October–April mean total rainfall: 47.30 cm) [25] and extensive irrigated agriculture, especially rice and row crops, such as tomatoes. SYMVCD, established in 1946 to protect the public from nuisance mosquito biting and mosquito-borne diseases, manages mosquito populations in Sacramento and Yolo counties [26].

### Aerial treatments and mosquito collections

SYMVCD provided the spatial polygons (Fig. 1b) and associated data detailing the date, area targeted for spraying, number of consecutive nights of spraying in the same location and pesticide product used for all aerial sprays during the study period (1021 unique nights of spraying during 930 spray events).

Mosquito collection records for CDC CO_2_-baited EVS traps [27] from SYMVCD for the period 2006–2017 (Fig. 1c) were obtained with permission through the CalSurv Gateway [28], an online database hosting data from California vector control agencies. Any records that indicated trap malfunctions or which ran longer than 1 night were excluded. Each record (*n* = 24,344) contained latitude, longitude, date, number of traps employed and total female *Cx. tarsalis* and *Cx. pipiens* captured. The distribution of traps and spray events by year (Additional file 1: Figure S1) and season (Additional file 2: Figure S2) are presented in the Supplementary Information.

Any records that indicated trap malfunctions or traps which were operated for > 1 night were excluded. For each species separately, we removed the collection reports corresponding to those > 2 standard deviations above the mean in each week to remove the influence of outliers (i.e. large singular deviations from broader abundance trends) on smooth functions subsequently estimated by the models.

All geographic data were projected from geographic to planar coordinates (Albers conic equal-area; EPSG 3310, NAD83) using the* rgdal* package in R statistical software (version 3.3.2; [29, 30]) for all data processing and analysis.

### Covariate development

To isolate the effects of aerial insecticide treatments within our final statistical model, we first developed a set of spatio-temporal and environmental covariates to explain the long- and short-term trends in *Cx. tarsalis* and *Cx. pipiens* abundance. Inclusion of these covariates established a counterfactual basis in the models for the expectation in abundance in the absence of controls, leaving the additional terms characterizing aerial insecticide sprays to explain any deviations attributable to the treatments.

For each remaining trap collection (*n* = 23,707 for *Cx. pipiens*; *n* = 23,678 for *Cx. tarsalis*), we derived a set of temperature variables to capture the effect of weather on trap collections. The mean temperature during the host-seeking period (dusk to dawn) and 30-year monthly average temperature were determined for each collection using 4-km resolution data provided by the PRISM Climate Group [31]. We calculated the deviation in temperature from the monthly average during the host-seeking period to capture activity rates on the night of trapping (i.e. warmer/colder than ‘normal’ resulting in higher/lower mosquito activity and resulting trap counts). As mosquito developmental rates are highly impacted by temperature [32, 33], we also calculated the average temperature during the 2-week period immediately preceding the trap collection to capture the short-term effects of weather on mosquito abundance. Rainfall was not considered because amounts were negligible in the study area during the season when aerial insecticide applications occurred.

To characterize the larval habitat present around a trap location and to incorporate the sharp changes in land use across the study area, we used the 30 × 30-m gridded land cover data from the 2011 National Land Cover database [34]. We used the classification of all pixels within a 5-km radius area surrounding each trap to determine the proportion of three non-overlapping land use categories: urban, cultivated crops and natural (Fig. 1a). Land use categories were chosen to represent larval habitat and bionomics of *Cx. pipiens* and *Cx. tarsalis* [35]. The radius was chosen based on known dispersal distances for the species [36, 37]. The ‘urban’ category encompasses all levels of developed land (i.e. structures, roads and constructed materials). The ‘crops’ category represents annual irrigated crops, of which rice is the predominant type in the study area. The remaining classifications were combined to create the ‘natural’ category.

We quantified the spatio-temporal intersections between spray zone polygons and trap locations to quantify the degree to which antecedent spray events impacted mosquito collections. Spatial coverage of each trap was quantified as the average proportion of the area from which a trap collects mosquitoes (‘collection area’) that was covered by aerial treatment zones during the 4 weeks preceding the collection. Using a conservative estimate on *Culex* flight distance [36–39] and to account for insecticide drift during application, we used a 5-km radius collection area for both species. The temporal sequence of overlapping sprays during the 4 weeks preceding a collection (modeled as a factor for each unique sequence) was used to capture any lagged effects and impacts of repeated spray events. For each week preceding a collection, the total proportion of the collection area overlapping with the treatment zone was assessed. When multiple treatment zones overlapped with a single collection area in a week, we assumed an additive effect, summing the proportions of overlap from each unique spray up to a maximum of 1.0 that represented complete coverage. We used the average spatial coverage of targeted spray zones during all weeks with at least one overlapping spray event to quantify the spatial impact of sprays for each specified temporal sequence of spraying. Traps > 5 km from all treatment zones had a spatial coverage of zero and corresponded to the reference level of the temporal sequence factor. These traps were included to capture baseline spatio-temporal mosquito dynamics in the absence of aerial treatments. Therefore, the impact of aerial spray events on collections was quantified with a twofold approach, namely with the factor corresponding to the sequence of weeks when spraying overlapped during the preceding 4 weeks and the average proportion of the collection area that overlapped under that sequence.

According to guidelines from the California Department of Public Health, periods of high risk for arbovirus transmission are characterized in part by abnormally high mosquito abundance [12]. In order to capture this dramatic deviation from ‘normal’ abundance that our smoothed modeling framework could not capture, but which precipitated aerial spray events, we also applied a prospective assessment to identify spray events closely following each collection. To account for the time required to respond to a high-risk period, we assessed the presence of overlapping treatment zones with a collection in the following 4 weeks on the weekly scale, similar to the above retrospective assessment of sprays.

To capture potential differences between broad classes of pesticides used (organophosphate* vs* pyrethrin and pyrethroids combined), we included a binary indicator variable for whether at least one spray event associated with a particular trap collection used an organophosphate. Sample sizes were too small to further investigate differences between pyrethrins and pyrethroids or between individual insecticide products.

We considered time in a variety of ways to capture trends in mosquito abundance in two parts: typical annual seasonality and coarser spatio-temporal trend over the 12-year study period. Using trap collection dates, ‘week’ (number of weeks from the start of the study period; range 1–626) and ‘day’ (range 1–365), variables were created to capture a continuous yearly effect and seasonality, respectively. We interacted the ‘day’ variable with each category of land use (i.e. ‘urban,’ ‘crops’ and ‘natural’) to capture the seasonal trend in these different habitats. Each individual seasonality curve was weighted by the proportion of land use in that category within 5 km of the trap collection to produce a single unified seasonal trend that reflected the specific habitat composition for that collection.

### Statistical analysis

We developed GAMs to relate nightly trap counts of female mosquitoes, either *Cx. tarsalis* or *Cx. pipiens*, to aerial adulticide applications, adjusted for variation in trap counts due to spatio-temporal mosquito dynamics. We chose GAMs because of the flexible parameterization of smooth functions of covariates to explain spatial and temporal trends [40, 41]. Covariates considered to explain baseline mosquito dynamics were day or week of the year, year, location, land use, 2-week average temperature and nightly deviations from average temperature during trapping, the presence of a spray event in the following 1 to 4 weeks (high-risk period) and pesticide class used in the aerial spray. Either a smooth function or a factor was used in fitting the covariates, with the choice between these forms, along with the spline and basis dimension if a smooth function was chosen, based on model fit and biological relevance. Cyclic cubic regression splines were used to prevent discontinuity between the ends of the smooth representing the seasonal patterns (i.e. between 31 December and 1 January). Thin plate regression splines were chosen for most covariates because they are isotropic and have been shown to be the optimal smoother of any given basis dimension [42]. A cubic regression spline was used in the spatio-temporal surface due to its superior performance over thin plate regression splines for the large amount of observations [43].

We fit negative binomial GAMs using the *gam* function in R (version 3.3.2; package *mgcv*) [29, 44] with restricted estimation maximum likelihood (REML) as the smoothing parameter estimation method. We chose a negative binomial function to account for the over-dispersed nature of the trap count data and used backward selection guided by the Akaike information criterion (AIC) [45] to reach our final model. In each model, we included an offset term for the number of traps operated per trapping event. All covariates were included in the initial model, and choices of interactions between covariates entered into the initial model were guided by biological relevance. We used concurvity, a measure of collinearity for smooth functions (range 0–1; 43), and visually examined deviance residuals for consistency in space and time to assess the final model fit.

Using the other covariates to establish the expected abundance of each species in the absence of aerial spraying, we estimated the mean change in predicted abundance across the range of spray regimes observed in the data, using the Bayesian posterior covariance matrix for the parameters that accounted for smoothing parameter uncertainty [43]. We simulated 10,000 random draws from the posterior distribution of the fitted model, a multivariate normal distribution with mean equal to the estimated model coefficients and covariance matrix of the parameters, to predict the abundance of each species across the spatial and temporal sequences of sprays observed in the data. For each draw, we then calculated the mean change in abundance from the baseline no-spray scenario at each point on the spatio-temporal surface, along with the corresponding 95% confidence interval (CI). Estimates of efficacy from the model were compared with those derived from Mulla’s formula [16, 17].

An R script outlining the workflow of parameter development, model fitting and estimating change in abundance across the spatio-temporal surface is presented in Additional file 3: Text S1.

## Results

### Data overview and model selection

**Fig. 2:**
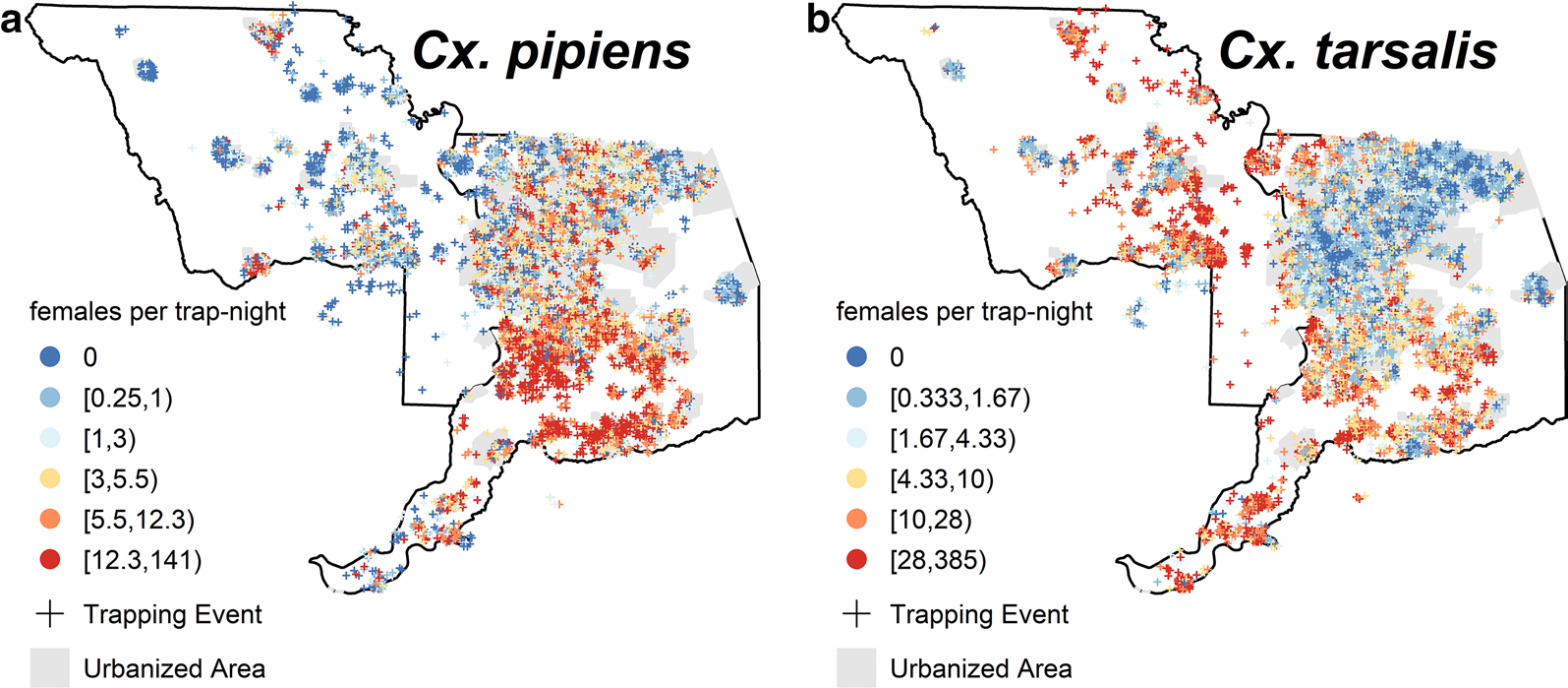
Collections of *Culex pipiens* (**a**) and *Cx. tarsalis* (**b**) during peak West Nile virus (WNV) season. Plus signs (*+*) indicate the location and number of female mosquitoes per trap-night for each collection during the period when aerial spraying occurred (late June to early October). Colors represent abundance quintiles by species for non-zero collections. A random shift of ≤ 1 km was applied to trap locations to aid visualization of repeated collections at the same locations during the study time frame

The relative abundance (number of females per trap-night) of *Cx. pipiens* and *Cx. tarsalis* varied spatially during the peak WNV season from late June to early October when aerial sprays occurred (Fig. 2). Typically, higher abundance of *Cx. pipiens* was observed in urban areas, whereas higher abundance of *Cx. tarsalis* was typically in non-urbanized areas near irrigated agriculture.

**Fig. 3:**
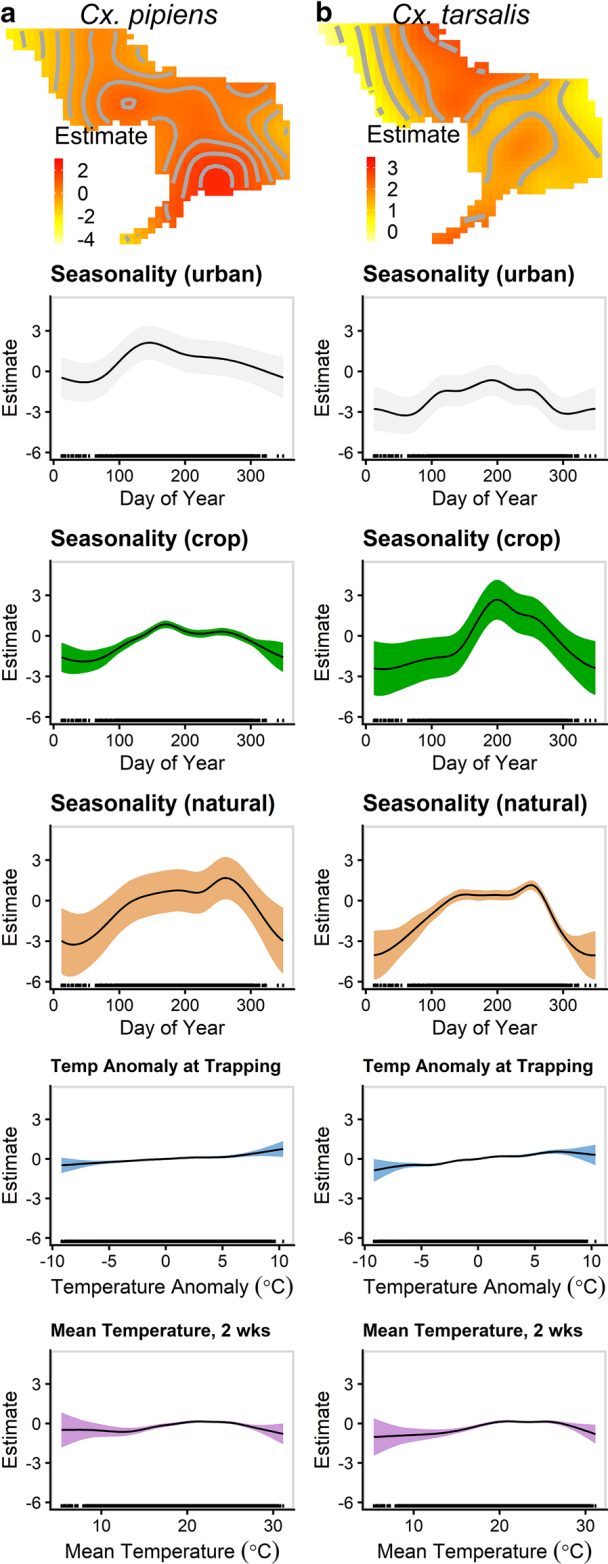
Smooth covariate functions explaining nightly abundance of *Cx. pipiens* (**a**) and *Cx. tarsalis* (**b**). Smooth functions from final generalized additive models are shown for the spatio-temporal surface (top), seasonality in a fully urban area, seasonality in a fully crop area, seasonality in a fully natural area, deviation (°C) from the 30-year monthly average temperature on the night of trapping and the average temperature (°C) during the 2 weeks prior to trapping (bottom). The shaded region represents the 95% confidence interval for one-dimensional functions. A representative slice of the three-dimensional spatio-temporal surface is presented for 2011 at the midpoint of the typical WNV season (week of 1 August). Spatio-temporal surfaces are plotted on individual axes for each species to resolve the spatial scale

The final model for each species included an offset for the number of traps run per collection event, and smooth functions of space by time (on the weekly timescale across the 12 years of the study), day of the year by each land use category (‘urban,’ ‘crops’ and ‘natural’), 2-week average temperature, nightly deviations in average temperature during trapping and spatio-temporal impacts of aerial spraying. Our choice of cutoff for removing outliers during model fitting did not significantly change our results. Removing the top 0, 4.5 or 10% of data in each week for each species resulted in only minor shifts in confidence interval widths and magnitude of some estimates, but no change to inference. The resulting smooth functions for each species are illustrated in Fig. 3 (for spatio-temporal surfaces for all years for each species, see Additional file 4: Figure S3; Additional file 5: Figure S4). The construction of smooth functions used in the final models is outlined in Additional file 6: Table S1. All smooth functions were highly significant (*P* < 0.0001). A random intercept for site location was included to account for repeated collections at the same location, fitted using coefficients penalized by a ridge penalty [46]. We also retained, based on reductions in AIC, parameters for the presence of sprays in the 1 & 4 and 1, 2, & 3 weeks following the trap collection for *Cx. tarsalis* and *Cx. pipiens*, respectively (Additional file 7: Table S2). Based on reduction in AIC, only the model for *Cx. pipiens* retained the term indicating the presence of at least one spray event with an organophosphate pesticide during the previous 4 weeks, as compared to all sprays using combinations of pyrethrin or pyrethroid products (− 48.9% change in abundance for ≥ 1 organophosphate; *P* < 0.001).

The largest magnitude of variability in the baseline abundance for both species was due primarily to the seasonality covariates, followed by the spatio-temporal surface. The temperature covariates contributed the smallest magnitude to establishing abundance, but all smoothed functions were highly significant (*P* < 0.0001). In all smooth functions, positive estimates correspond to increases in the population, negative estimates correspond to decreases in the population and 0 indicates no modulation in abundance at that covariate value.

The distribution of the final model residuals was right-skewed for both species, indicating the model underestimated extreme trap counts. However, no spatial or temporal pattern remained in the deviance residuals (Additional file 8: Figure S5; Additional file 9: Figure S6). Both models had low estimated concurvity values [43] for the aerial spraying smooth function with the rest of the model parameters (*Cx. pipiens*: 0.145; *Cx. tarsalis*: 0.148), indicating that the smooth estimates for the impact of aerial spraying were not confounded by other parameters. In addition, the relatively high deviance-explained value for both models (*Cx. pipiens*: 44.0%; *Cx. tarsalis*: 62.3%) indicates good model fit despite the complex dynamics inherent in mosquito populations.

### Effects of aerial insecticide treatments

**Fig. 4:**
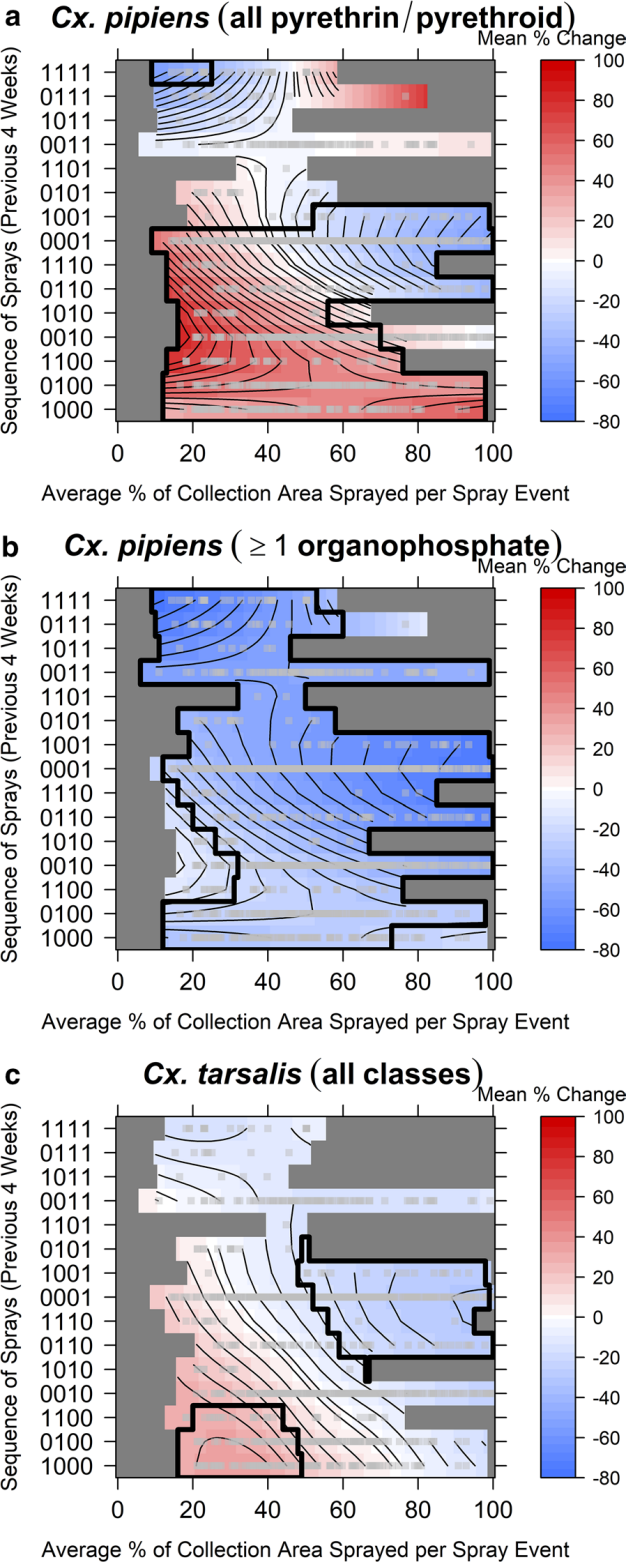
Mean changes in abundance following aerial spraying, as compared to no-spray baseline. Estimates are shown for changes in abundance of *Cx. pipiens* (**a**, **b**) and *Cx. tarsalis* (**c**) with respect to antecedent sequence and average spatial coverage of aerial treatments. **a**, **b** For *Cx. pipiens*, estimates are shown for sprays that used only pyrethrin or pyrethroid products (**a**) and for sprays that utilized an organophosphate product at least once (**b**). **c** For *Cx. tarsalis*, estimates are with any product class. Horizontal axes represent the average proportion of the 5-km buffer surrounding a trap covered by a spray event, and vertical axes represent the temporal sequence of aerial sprays during the 4 weeks preceding the trapping event. On the left vertical axis, the 4-digit sequence indicates presence (*1*) or absence (*0*) of sprays in the 1, 2, 3 and 4 weeks (going from right to left along sequence) prior to trapping. The sequences of spray events are ordered from the fewest number and temporally most distant spray events (bottom) to the largest number and temporally closest spray events (top). Estimates are truncated to the range observed with the available data (gray squares indicate points present in dataset). Areas enclosed in a black border represent the portion of the spatio-temporal surface with significant estimates (*P* < 0.05)

A smooth surface of the spatio-temporal impacts on *Cx. pipiens* abundance is presented for treatments with only pyrethrin or pyrethroid products (Fig. 4a) or with at least one organophosphate product (Fig. 4b). For *Cx. tarsalis*, the difference in impact by broad pesticide class was not retained in the final model, and therefore a single smooth is presented (Fig. 4c). Overall, the models estimated a lower magnitude of change in *Cx. tarsalis* abundance as compared to that in *Cx. pipiens* abundance. For example, following aerial spraying with full spatial coverage (i.e. 100% coverage of the area within 5 km of the trap), we estimated a mean 1-week change in *Cx. pipiens* abundance of − 52.4% (95% CI − 65.6, − 36.5%) if all spray events had used pyrethroid or pyrethrin products. If at least one organophosphate product had been used, we estimated a mean change in *Cx. pipiens* abundance of − 76.2% (95% CI − 82.8, − 67.9%). In contrast, *Cx. tarsalis* populations with full spatial coverage by aerial sprays showed an estimated mean 1-week post-spraying change in abundance of − 30.7% (95% CI − 54.5, 2.5%) regardless of the product class.

For both species, larger reductions in abundances were estimated in areas with higher spatial coverage of aerial sprays (large proportion of spatial overlap) than those on the fringes (low proportion of spatial overlap). Sprays occurring closer in time to collections were generally estimated to result in larger reductions in abundance compared to those that occurred further back in the past. At longer time lags (i.e. 2–4 weeks post-spraying), higher than expected abundance for both species was estimated, with the increase only occurring in *Cx. pipiens* populations following sprays with pyrethrins and pyrethroids.

The majority of temporal spray sequences lacked data across the full range of spatial overlap (0–100%); we did not estimate the change in abundance for these areas. Data were sparser or lacking for higher spatial coverage during multiple weeks of spraying. For regions of the spatio-temporal surface with data support, the reduction at 1-week post-spraying with full spatial coverage was the largest reduction predicted for *Cx. tarsalis*. In contrast, results for *Cx. pipiens* indicated a similar reduction for populations on the fringes of spray events for the preceding 4 weeks and populations with full spatial coverage by aerial sprays 1 week previously (change all pyrethrin/pyrethroids: − 54.3%; 95% CI − 81.0, − 6.2%; change at least one organophosphate: − 77.2%; 95% CI − 90.4, − 53.9%).

### Comparison to conventional estimates

As a comparison for our model results, we applied the conventional approach of Mulla’s formula [16, 17] to the combined trapping and control records from 2006 to 2017 to estimate the efficacy of sprays. Considering trapping 1 week before and 1 week after a spray event and using a 5-km buffer around the targeted spray zone as the adjacent comparison area, we were able to calculate the effect for 36 spray events (3.87%) for *Cx. pipiens* and *Cx. tarsalis*; the majority of spray events lacked traps in all of the required spatial and temporal locations for the calculation. Most of the estimates for the change in abundance indicated a reduction in trap counts, but estimates varied widely, ranging from complete population elimination (100% decrease) up to increased of 11,000% following a spray event (Additional file 10: Figure S7). Most estimates for *Cx. pipiens* indicated varying degrees of reduction while those for *Cx. tarsalis* spanned reductions to increases.

## Discussion

This study found that aerial insecticide treatments achieve strong short-term reductions in both *Cx. tarsalis* and *Cx. pipiens* populations. Previous studies have assessed the short-term impact of aerial spraying on mosquito abundance and highlighted the volatility of estimates across space and time. In order to overcome the limitations of using single events to estimate the efficacy of aerial spraying on reducing the abundance of WNV vector mosquitoes, we used a large dataset of surveillance and control records together with GAM models. This modeling framework allowed us to establish baseline mosquito adult abundance and identify deviations from expected nightly abundance attributed to aerial spraying (i.e. counterfactual basis) as well as the spatial and temporal impacts of aerial applications. Our results indicate that aerial sprays do achieve population reduction for both *Cx. pipiens* and *Cx. tarsalis* with heterogeneity in the magnitude and pattern of reduction between species and pesticide product.

The differences in the magnitude and pattern of the estimated response between species can be attributed partially to the different bionomics of the individual mosquito species. In the study area, *Cx. pipiens* are predominantly peridomestic with larval habitats limited primarily to backyard sources and stormwater systems in urbanized areas [35, 47]. Thus, areas targeted by aerial insecticide treatments would span a large fraction of any particular population, leaving few adults to repopulate the treated area from proximal unsprayed locations. In contrast, *Cx. tarsalis* breed in agricultural areas and may disperse into surrounding agricultural and urban areas [35, 48, 49]. This species also has a larger typical dispersal distance and achieves higher population densities than *Cx. pipiens* [35–37]. Aerial insecticide treatments typically target only a small fraction of the total available habitat for *Cx. tarsalis*, often habitats near urbanized areas, and any effect of aerial sprays could be moderated by immigration of adult *Cx. tarsalis* from surrounding unsprayed locations, potentially from distant locations [38, 50]. Therefore, the best suppression of *Cx. tarsalis* populations would be achieved in isolated areas, as has been reported previously [22]. Additionally, repetition of sprays on the weekly scale is less effective at controlling *Cx. tarsalis* than *Cx. pipiens* because of the rapid immigration and emergence of new adults from large areas of productive larval habitat.

While our estimated reduction in the abundance of *Culex* mosquitoes shows some similarity to previous published estimates of aerial spray events from Sacramento and Yolo counties (Table 1), our methodology also accounts for contextual factors, resulting in more robust estimates of the average effect of spraying. Previous estimates exhibited spatial heterogeneity [19]. Utilizing covariates to capture the spatial structure, temperature deviations and varying distribution of larval habitats removed the confounding impact of these factors on our model results. Similarly, previous estimates have varied, in part because Mulla’s formula cannot fully capture spatio-temporal nuances of mosquito population dynamics. For example, Lothrop et al. [20] observed 73% increases in *Cx. tarsalis* abundances post-spraying despite observing mortality in caged sentinel mosquitoes and large reductions during previous spray events. The authors attributed the estimated increase to the dynamics of *Cx. tarsalis* populations at the time of the study, particularly the large emergence of *Cx. tarsalis* following the annual flooding of the nearby wetlands that was not captured by the Mulla’s formula framework and the fact that the sprays were not impacting mosquitoes in the productive larval habitats. Previous estimates also used differing time interval lengths to estimate mosquito abundance before and after spray events. The heterogeneity in these time intervals combined with the heterogeneity of the resulting estimates (Table 1) highlight the need to use consistent time intervals to improve generalizability of estimates between studies. As standardization of the time interval largely depends on the operational capacity of vector control districts for trapping and responding to epidemic conditions, no single recommendation may be feasible across all studies. However, we recommend that mosquito control agencies should keep the time frame consistent across their evaluations to increase comparability of intra-agency control efforts. A similar range of estimates for change in *Culex* abundance following adulticide treatments has been reported outside California; most are in broad agreement with our findings, although none used Mulla’s formula. In Chicago (Illinois), a reduction of 54% in *Cx. pipiens* trap counts within the spray zone* versus* the baseline pre-spray abundance was reported, in contrast with a 153% increase outside the spray zone, following two single-night aerial spray events with a pyrethroid 7 days apart [51]. An average 65.3% reduction in *Cx. pipiens*/*restuans* populations was observed within 24 h of truck-mounted applications of a pyrethroid [52]; in contrast, no significant changes in *Cx. pipiens* abundance were observed following single-night, truck-mounted applications of a pyrethroid in three communities near Boston (Massachusetts) [53]. Up to a 75% reduction in the 2-day counts of female *Cx. quinquefasciatus* was reported during a month-long period with truck-mounted pyrethroid sprayed 5 days a week in Dubai, United Arab Emirates [54]. Without untreated comparison locations, it is hard to directly compare these results to those of our study.

**Table 1:** Estimated change in *Culex* mosquito populations following aerial spray events in California using Mulla’s formula

Location in California (city, county)	Year	Nights sprayed^a^	Product class	Comparison length of time^b^	Species	% Change^c^	Reference
Davis, Yolo	2006	2	Pyrethrin	2 days before, 2 days after	*Cx. pipiens*	− 58.0	[19]
					*Cx. tarsalis*	− 25.6	
Woodland, Yolo	2006	2	Pyrethrin	2 days before, 2 days after	*Cx. pipiens*	− 77.7	
					*Cx. tarsalis*	− 46.8	
Sacramento, Sacramento	2005	3	Pyrethrin	7 days before, 7 days after	*Cx. pipiens*	− 75.0	[21]
					*Cx. tarsalis*	− 48.7	
Sacramento, Sacramento	2006	3	Pyrethrin	3 days before, 3 days after	*Cx. pipiens*	− 39.3	[85]
					*Cx. tarsalis*	− 57.3	
Coachella valley, Riverside	2005 (March)	3 alternate^d^	Pyrethroid	5 days before, 1 day after	*Cx. tarsalis*	− 93.0	[20]
	2005 (June)	3 alternate^d^	Pyrethroid	5 days before, 1 day after	*Cx. tarsalis*	− 77.0	
	2005 (September)	3 alternate^d^	Pyrethroid	5 days before, 1 day after	*Cx. tarsalis*	73.0	

^a^Number of consecutive nights sprayed in a spray event

^b^Length of time before and after an aerial spray event used when comparing with trap counts

^c^Percentage change in* Culex* abundance following an aerial spray event, as calculated using Mulla’s formula [16, 17]

^d^Aerial spraying occurred on 3 alternate nights

Our model structure most closely resembles the study design used by Elnaiem et al. [21] where a timescale of 1 week before and after a spray event with a pyrethroid pesticide was chosen when assessing mosquito abundance (Table 1). Our estimates for reduction for *Cx. pipiens* (− 52.4%) and *Cx. tarsalis* (− 30.7%) are lower than the observed reductions (*Cx. pipiens*: − 75%; *Cx. tarsalis*: − 48.7%). While qualitatively similar, the differences in magnitude may be due to differences in analytical methods, shifts from pyrethrin to pyrethroids over time or the longer 12-year time period of our study that could have yielded a more conservative estimate of average spray effects.

Our approach to causal inference using observational data builds on earlier contributions from the fields of environmental science and epidemiology. Mulla’s formula can be considered to be an extension of the Before–After–Control–Impact (BACI) analysis framework. Originally defined by Green [55] and extended and applied by others [56–59], the BACI analysis framework originated in environmental science literature to distinguish natural variability from the impact of an anthropogenic disturbance, and has been applied to mosquito larvicide evaluations [60, 61]. The BACI methodology compares an impact and at least one separate control location, sampled at various time points before and after the impact, to detect changes in the natural history of the environment due to the impacts [56–58]. An analysis of variance (ANOVA) test is used to detect a significant difference in the trajectories before* versus* after the disturbance in the impact area as compared to the control area. The location and timing of sampling in each trajectory is chosen to ensure each is independent across space and time and increase the evidence that a detected change was attributable to the disturbance itself [56, 59]. Our GAM framework extends BACI using a three-dimensional spatio-temporal function and other covariates to capture the entire spatio-temporal context as a strategy to estimate the expected mosquito abundance in the absence of spraying. The functions also capture trends in the impact over spatial and temporal combinations, while the BACI framework may miss significant changes due to the sampling time frame chosen [62]. Additionally, our methods do not depend on the ANOVA assumptions of independence and homoscedasticity of samples [63], as the spatial and temporal correlation and the non-normal distribution of trap collections are accounted for through the covariates in the negative binomial GAMs.

Our statistical approach for estimating the effects of mosquito control on abundance relies on counterfactual theory that has been applied in the field of epidemiology as a conceptual basis for understanding measures of effect [64–66]. Counterfactual theory as a basis for causal inference is premised on the assumption that for any unit being observed, there are multiple potential exposures but only one actually occurs, and outcomes under other alternative exposures exist only as potential outcomes that would have occurred if an alternative exposure had been applied. Because the alternative exposures did not occur, these are contrary to fact, or counterfactual. In this study, our units of study were trapping locations, and we sought to understand the effect of aerial spraying by statistically relating the observed mosquito abundance following spray events to the mosquito abundance in the same place and time that would have been observed in the absence of the spraying. BACI and Mulla’s formula approaches utilize untreated control sites to establish expectations for the unsprayed condition. Our approach instead aims to estimate the counterfactual expectation for mosquito abundance directly at the same place and time using spatio-temporal trends and contextual variables (i.e. weather and land use). This approach offers two clear advantages for estimating the effects of public health pesticide use: (i) it allows for use of rich observational datasets that already exist and which capture pesticide usage in real operational contexts, as opposed to experimental settings that are often closer to ideal conditions; and (ii) it does not rely on pre-selected, untreated control sites, which is helpful because vector management programs are rarely willing to withhold treatments in experimental control sites if their public health action thresholds are met.

The smooth functions associated with land use categories in the final GAMs accurately captured known seasonal and population dynamics. Cultivated crops were the primary source of *Cx. tarsalis*, with smaller contributions from other, non-urban land types during the warmest months of the year, accurately representing the presence of highly productive larval habitats in clean, recently created water sources characteristic of cultivated crops [35, 48, 67]. Urbanized areas did not produce large numbers of *Cx. tarsalis* during the WNV season as they contain few suitable larval habitats for this species. The estimated peak in abundance occurred in late July, but remained high through September, capturing the variation in timing of the peak across the years of the study. Populations of *Cx. tarsalis* in the Sacramento Valley are greatest from July to September [35, 68, 69]. The steep slope of the curve up to the peak mimicked the rapid increase in *Cx. tarsalis* observed at the start of the planting season [35, 69]. For *Cx. pipiens*, urbanized areas largely contributed to abundance throughout the year, with additional contributions in natural areas (i.e. non-cultivated croplands) later in the season, reflecting the presence of larval habitats in artificial structures like storm drains or dairy wastewater lagoons [70, 71]. Crops were generally associated with lower *Cx. pipiens* abundance across the season, reflecting the general lack of suitable high-quality larval habits in these areas.

Temperature anomalies during trapping and the 2-week average antecedent temperature prior to trapping contributed to the overall abundance of both species, albeit relatively weakly in the presence of the other spatio-temporal terms. Their inclusion in the model was required to fully account for mosquito dynamics and night-to-night fluctuations in trapping success. Concordant with previous experiments, extremes in the average temperatures reduced abundance for both species, illustrating the negative impacts on mosquito developmental rates and adult survivorship [32, 33]. The estimated region of positive contribution to abundance for both species (*Cx. pipiens*: 19.2–25.4 °C;* Cx. tarsalis*: 18.9–27.0 °C) was narrower than the thermal tolerance of the species, but it contains the observed regions of rapid developmental and high reproduction rates and the typical temperature ranges during the summer. As expected, small anomalies in average temperature on the night of trapping made relatively small contributions to change in the abundance while extreme deviations resulted in much more marked change, highlighting the non-linear relationship underlying temperature and trap success.

It is interesting to note the additional marked reduction in *Cx. pipiens* abundance when at least one organophosphate product was used, especially as compared to the lack of a similar difference in *Cx. tarsalis* populations. As mentioned in the preceding text, the class of product used may be more important for *Cx. pipiens* due to their focal distributions and more limited dispersal [35, 72]. As an aerial spray will likely impact a large proportion of the localized *Cx. pipiens* population at once, there will be limited immigration from unsprayed segments of the population in the nearby proximity. Therefore, the full effect of an aerial spray is discernable. In contrast, the dispersed nature of *Cx. tarsalis* populations facilitates rapid immigration from surrounding unsprayed locations [35, 36, 38], thus diluting any difference in effect between product classes; any difference is not discernable against the background population dynamics accounted for in our modeling framework. Another factor contributing to the difference by species could be insecticide resistance, as resistance to pyrethroids and organophosphates has been reported for both species in California [14, 73]. If *Cx. pipiens* populations in the study area were more resistant to pyrethroids than *Cx. tarsalis*, as has been previously reported in the Central Valley [74–76], this could explain the increased efficacy of organophosphates for *Cx. pipiens*. However, since we found a stronger effect of pyrethroids on *Cx. pipiens* as compared to *Cx. tarsalis*, resistance does not fully explain the observed difference. Alternatively, a single organophosphate spray may be insufficient to produce a marked difference in *Cx. tarsalis* populations; a repetition may be required. The underlying shape of the smooth function of spatio-temporal impacts of aerial spraying for either species likely differs between product classes and the specific timing and number of different products used, but sparse data prevented us from including an interaction to assess these dynamics.

A potential population rebound effect occurred for both species at more distant time lags from spraying in cases where abundance was estimated to be higher than expected at 2–4 (*Cx. pipiens* under pyrethrin and pyrethroid sprays) and 3–4 (*Cx. tarsalis*) weeks post-spray. Appropriately spaced treatments in time may be required to maintain a long-term reduction in population abundance. However, such a rebound does not negate the potential value of aerial treatments for achieving short-term reductions in the abundance of WNV-infected adult mosquitoes during periods of epidemic risk.

The increase in abundance at low spatial coverages of sprays for both species could reflect excito-repellency of pesticides at the fringes of targeted areas. Excito-repellency, a form of behavioral avoidance, combines two forms of sub-lethal exposure that results in mosquito movement away from a chemical source, namely contact excitation (increased activity upon contact) and non-contact spatial repellency [77–79]. These non-toxic behavioral impacts of pesticides were first identified in *Anopheles* mosquitoes in response to DDT and later with insecticide-treated bednets and indoor residual spraying [80–82]. Populations of *Cx. quinquefasciatus*, another species in the *Cx. pipiens* complex, exhibit strong contact excitation and poor spatial repellency to pyrethroid, organophosphate and carbamate pesticides [83, 84]. No study has investigated excito-repellency in *Cx. tarsalis* populations. The behavioral avoidance of *Culex* to pesticides could be pushing mosquitoes out of the spray zones, resulting in an increase in abundance around the fringes of sprays, consistent with our estimates for spatial coverages of < 40% for *Cx. pipiens* following sprays with pyrethrin or pyrethroids only in the previous week.

A limitation of choosing a GAM framework is that we were only able to capture the average effects of covariates on nightly mosquito trap counts and unable to fully account for large stochastic fluctuations inherent to mosquito populations. However, GAMs easily allowed us to incorporate nonlinear relationships between abundance and covariates without having to constrain relationships with *a priori* knowledge. In particular, we were able to capture the higher-order relationships and correlation across space and time with the three-dimensional spatio-temporal function. The form of the smooth relationships in the final model did appear to approximate what is observed in nature. Other strengths of our modeling approach are that it takes into account regional differences in mosquito populations, population dynamics and seasonality in different land use types, as well as the impacts of short-term (night) and longer-term (2 week) weather, resulting in robust estimation of the baseline expected abundance in the absence of spray effects, allowing us to isolate the deviations in abundance due to aerial spraying. This counterfactual basis of the model enables estimation in the absence of an independent control. However, even with the large amount of data available, data were still inadequate to estimate the impact of aerial spraying reliably for certain time lags or spatial coverages that were rare or absent in the data. This is primarily due to typical SYMVCD spraying and trapping practices due to logistical and financial constraints. SYMVCD concentrated their mosquito collection efforts near urban areas to maximize the sensitivity for assessing the risk in proximity to human populations while minimizing time and costs associated with large-scale mosquito surveillance. Additionally, in an effort to control mosquitoes in known problem areas (highly productive larval habitats in proximity to the margins of urban areas) and reduce aerial applications over urban areas, the majority of areas receiving repeated sprays across the WNV season are in more rural areas where the mosquito trapping is sparser. This limited the data and the statistical power to quantify the full range of spatial overlap with aerial sprays.

We were also unable to account explicitly for drift outside the target zones during sprays, as has been previously described [20]. Our use of a continuous variable to measure spray coverage within the 5-km radius collection areas surrounding each trap partially accounts for this effect. As such, however, we are unable to fully parse out the effect of aerial spraying on populations outside aerial spray zones and limited our analysis to only assess spatial coverage of traps within targeted spray zones.

Additionally, we were unable to estimate the relative effects of different lengths of multi-night spray events (1* vs* 2* vs* 3 consecutive nights) due to data limitations and our analytical choice to aggregate all sprays on the weekly scale to achieve the balance between robust estimates and operationally relevant information for vector control districts. These limitations of observational studies such as this one could be addressed in future experimental field trials.

## Conclusions

Aerial adulticides were shown to achieve short-term reductions in the abundance of the primary WNV vectors *Cx. tarsalis* and *Cx. pipiens*. A greater reduction was estimated for *Cx. pipiens*, likely due to its focal distribution in urbanized areas and limited dispersal. The use of organophosphate products* versus* a combination of pyrethrins and pyrethroids increased the magnitude of reduction estimated for *Cx. pipiens* while the difference by broad insecticide class was not significant for *Cx. tarsalis*. The effects of aerial sprays on *Cx. tarsalis* populations were likely moderated by the broad dispersal ability of this species, its large population sizes and the vast expanses of productive larval habitat in the study area. Therefore, the best control of *Cx. tarsalis* would appear to be achieved in areas with isolated or highly spatially segmented populations. For both species, aerial spraying reduced abundance at high spatial coverage while reductions were also estimated at lower spatial coverage, at albeit greatly reduced magnitudes, indicating that aerial sprays had some impacts beyond the target zone. There was also evidence for population rebounds at periods of 2–4 weeks post-spraying. Our modeling approach allowed us to utilize observational data to isolate aerial treatment effects while taking into account contextual factors, such as spatio-temporal relationships, weather and habitat, that contribute to stochastic variation in nightly trap counts. This is an important advance that complements experimental trials and expands upon conventional observational approaches that summarize population changes following aerial treatments at individual time points. Further work should expand upon these methods to estimate the change in WNV transmission potential and resulting human infections following aerial spray events.

## Supplementary Information

**Additional file 1: Figure S1.** Location of CO_2_-baited mosquito trapping events and aerial spray events stratified by year (2006–2017). A random jitter of ≤ 1 km was applied to trapping locations for visualization of repeated events at the same site. Each spray event polygon represents the area targeted during a single aerial spray application.

**Additional file 2: Figure S2.** Location of CO_2_-baited mosquito trapping events and aerial spray events stratified by season. Season defined into three-month intervals. A random jitter of ≤ 1 km was applied to trapping locations for visualization of repeated events at the same site. Each spray event polygon represents the area targeted during a single aerial spray application.

**Additional file 3: Text S1.** R script outlining our workflow of covariate development, GAM model fitting and estimated change in abundance.

**Additional file 4: Figure S3.** Spatial surface for *Cx. pipiens* at midpoint of the typical WNV season (week of 1 Aug). Surface presented for each year (2006–2017) reflects the relative abundance of the species and is a slice from the three-dimensional spatio-temporal smoothed function. Contours applied for visualization of estimates.

**Additional file 5: Figure S4.** Spatial surface for *Cx. tarsalis* at midpoint of the typical WNV season (week of 1 Aug). Surface presented for each year (2006–2017) reflects the relative abundance of the species and is a slice from the three-dimensional spatio-temporal smoothed function. Contours applied for visualization of estimates.

**Additional file 6: Table S1.** Smooth functions included in the final GAMs for both *Cx. tarsalis* and *Cx. pipiens*. Table outlining the construction of smooth function used in the final models, including spline type and basis dimensions chosen.

**Additional file 7: Table S2.** Change (%) in nightly abundance from expected for collections preceding aerial spraying. Table indicating the final model estimates for change in expected trap-counts for collections in the 1 to 4 weeks preceding an aerial spray event.

**Additional file 8: Figure S5. **Spatial and temporal distribution of model deviance residuals for *Cx. pipiens *for 2006–2017. Residuals presented spatially at the associated trapping location (random jitter of ≤ 1 km was applied for visualization of repeated events).

**Additional file 9: Figure S6. **Spatial and temporal distribution of model deviance residuals for *Cx. tarsalis *for 2006–2017. Residuals presented spatially at the associated trapping location (random jitter of ≤ 1 km was applied for visualization of repeated events).

**Additional file 10**:** Figure S7. **Estimated percentage change in *Cx.* *pipiens *and *Cx. tarsalis *populations with Mulla’s formula. Change estimated for the 36 aerial sprays in Sacramento and Yolo counties, California (2006–2017) with associated trap collections within the targeted zone (treated) and an adjacent 5-km buffer (control) within 1 week before and 1 week following spraying.

## Data Availability

The data that support the findings of this study were obtained from the Sacramento-Yolo Mosquito and Vector Control District. These data were used with permission for the current study and are not publicly available. Data are however available from the authors upon reasonable request and with permission from the Sacramento-Yolo Mosquito and Vector Control District.

## Abbreviations

GAM: Generalized additive model
SYMVCD: Sacramento-Yolo Mosquito and Vector Control District
WNV: West Nile virus
