# Supplementary figures and images for: Spatio-temporal impacts of aerial adulticide applications on populations of West Nile virus vector mosquitoes
Source: Parasit Vectors. 2021 Feb 24;14:120. doi: 10.1186/s13071-021-04616-6 (PMC7905633; doi:10.1186/s13071-021-04616-6)

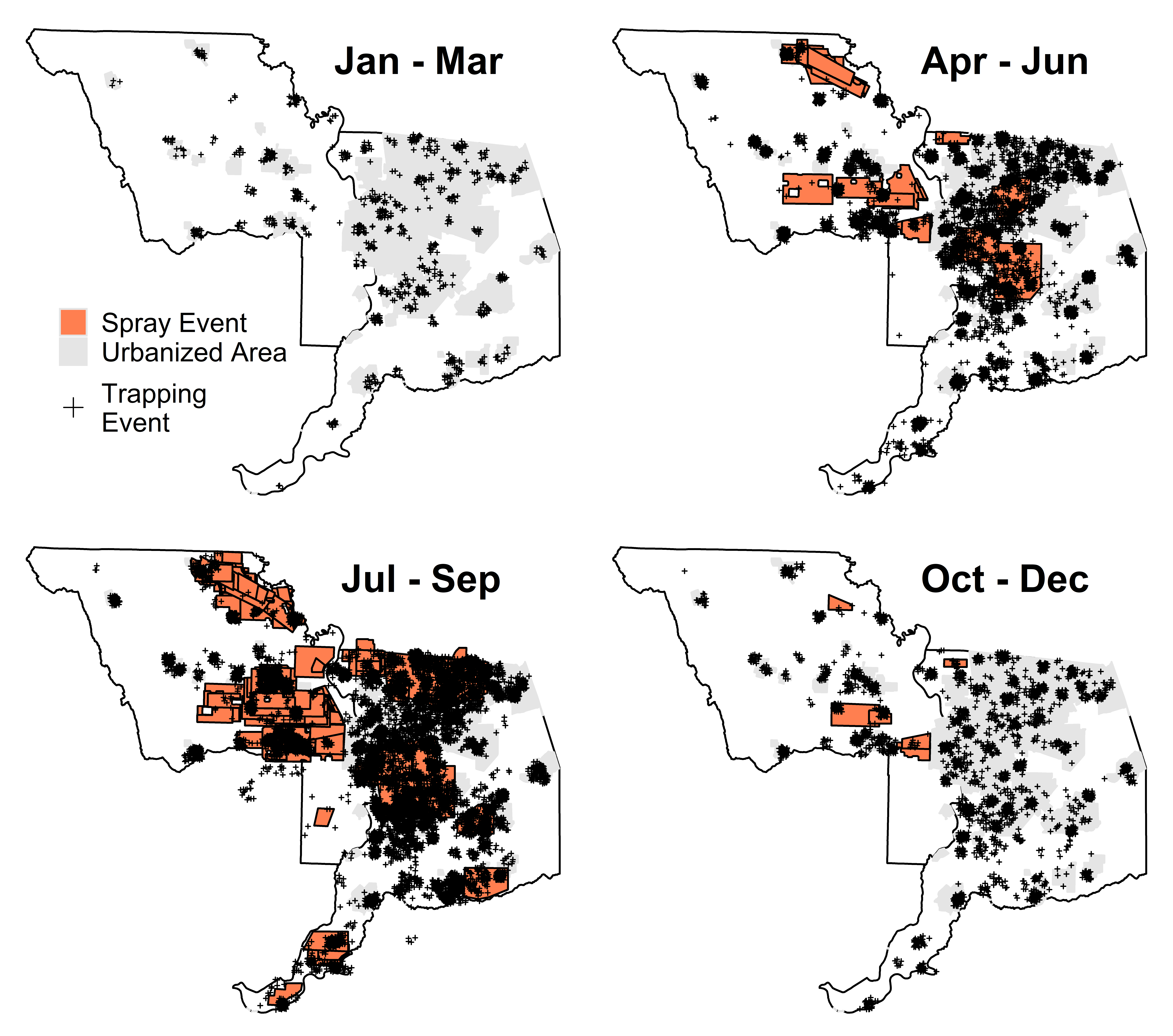

Supplement: Supplementary file 2 — Additional file 2: Figure S2. Location of CO2-baited mosquito trapping events and aerial spray events stratified by season. Season defined into three-month intervals. A random jitter of ≤ 1 km was applied to trapping locations for visualization of repeated events at the same site. Each spray event polygon represents the area targeted during a single aerial spray application. [file 13071_2021_4616_MOESM2_ESM.tif]

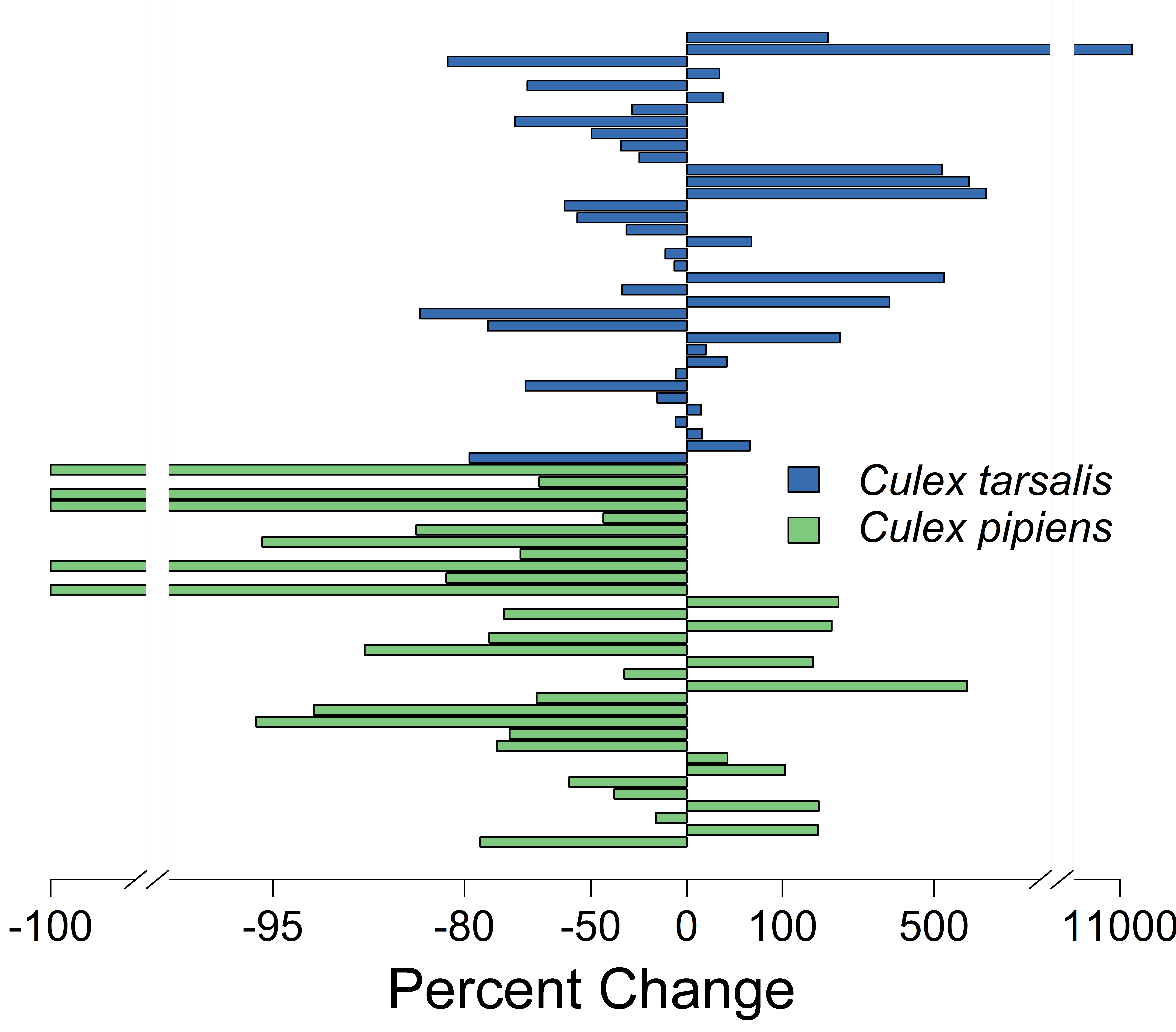

Supplement: Supplementary file 10 — Additional file 10: Figure S7. Estimated percentage change in Cx. pipiens and Cx. tarsalis populations with Mulla’s formula. Change estimated for the 36 aerial sprays in Sacramento and Yolo counties, California (2006–2017) with associated trap collections within the targeted zone (treated) and an adjacent 5-km buffer (control) within 1 week before and 1 week following spraying. [file 13071_2021_4616_MOESM10_ESM.tif]
