# Supplementary material for: Spatio-temporal impacts of aerial adulticide applications on populations of West Nile virus vector mosquitoes
Source: Parasit Vectors. 2021 Feb 24;14:120. doi: 10.1186/s13071-021-04616-6 (PMC7905633; doi:10.1186/s13071-021-04616-6)
Supplement: Supplementary file 3 — Additional file 3: Text S1. R script outlining our workflow of covariate development, GAM model fitting and estimated change in abundance. [file 13071_2021_4616_MOESM3_ESM.docx]

**Additional file 3**

**Text S1.** R script of GAM model fitting and estimation. Script outlines our workflow of covariate development, GAM model fitting, and estimated change in abundance. Code presented is not necessarily the most efficient, but provides the logic of our methodology to guide future use of this method.

#####

# Author: Karen Holcomb (kmholcomb@ucdavis.edu)

# Last Updated: 12/03/20

####

# Generalized version of workflow developed for estimation of the effect of aerial spraying

# on abundance of Culex tarsalis and Culex pipiens populations in Sacramento and Yolo county, CA

#

# Note: Code presented is compiled over many iterations and likely not the most efficient way

#

# Flow of code: pre-process data, spatial join of traps and spraying data, fit GAMs, estimate change in abundance

# Terminology of components:

# spray.spdf - Spatial Polygons DataFrame with shapefiles for each spray event and dataframe with

# information on each spray event (each row is a different spray; need column for date,

# name of spray event, and product used (code assumes 1 polygon per of row of spraying data)

# trap.data - dataframe with trapping record (each row is different trap event);

# need columns with date, number of traps involved per trapping event, lat/long, temperature,

# number mosquitoes collected, DiffSpray (see below)

# DiffSpray - number that identifies which sprays are part of the same multi-night spray event;

# same number means sprays are part of same event (my set-up method shown below)

####

## Set-up data frames (trap.data and spray.spdf) ################

library(mgcv) #GAM package

library(sp)

library(rgdal)

library(rgeos)

## Load the mosquito collection data (data.raw)

# Subset trapping data to CO2 traps run w/o issue; columns for long/lat, date of collection, number of traps

# run at that event, number of nights traps run, ID for collection location, total tarsalis and pipiens caught,

# and ID number for collection

Keep <- which(data.raw$trap_problem == "N" & data.raw$trap_type == "CO2")

trap.data <- data.raw[Keep,c("longitude","latitude","collection_date","num_trap","trap_nights",

"site_code","tarsalis_females","pipiens_females","collection_id")]

trap.data <- trap.data[trap.data$trap_nights == 1, ] #exclude any CO2 traps run longer than 1 night

## Set-up date-related variables for trap data ('collection_date' is date when trap was picked up)

trap.data$collection_date <- as.Date(trap.data$collection_date, format="%m/%d/%Y")

trap.data$Day <- strptime(trap.data$collection_date,format="%Y-%m-%d")$yday+1

trap.data$Year <- as.numeric(format(trap.data$collection_date,"%Y"))

trap.data$Week <- as.numeric(as.character(format(trap.data$collection_date, "%V"))) #week of the year

trap.data$YearWeek <- as.numeric(as.character(trap.data$Year)) + trap.data$Week/52 #continuous time

trap.data$Year <- as.factor(trap.data$Year)

## Set-up date-related variables for spray data ('date' is date aerial spray application occurred)

spay.spdf$date <- as.Date(spay.spdf$date, format="%m/%d/%Y")

spay.spdf$Day <- strptime(spay.spdf$date,format="%Y-%m-%d")$yday+1

spay.spdf$Year <- as.numeric(format(spay.spdf$date,"%Y"))

spay.spdf$Year <- as.factor(spay.spdf$Year)

## Set-up DiffSpray numbers to indicate which sprays are part of same multi-night spray event (based on name, date)

# assumes dates are oldest to newest

# 'name' is name of spray location in spray.data dataframe

diffy = vector() #holding vector for below loop, gathering info before putting all in new col together at end

for (i in 1:length(levels(spray.spdf$name))) {

ord <- cumsum(c(1, diff(spray.spdf$date[spray.spdf$name == levels(spray.spdf$name)[i]]) != 1))

loc <- which(spray.spdf$name == levels(spray.spdf$name)[i])

diffy[loc] <- as.numeric(paste(i)) + ((ord-1) / length(ord))

}

spray.spdf$DiffSpray <- diffy #diff spray events by diff numbers

## Add in temperature data as columns (used 2 week average and deviation from average on night of trapping)

# trap.data$av.temp_2wk #average temperature in 2 weeks prior to trapping

# trap.data$Temperature #deviation in temperature from average

# Used Daymet's Single Pixel Extraction tool: https://daymet.ornl.gov/single-pixel/) based on lat/long of traps

## Add in land use data (% of 5km buffer around trap of each land use type) from https://www.mrlc.gov/data/nlcd-2011-land-cover-conus-0

# Extract land use based on lat/long of trap w/ 5km buffer around each trap

# Land use categories (NLCD codes): urban (21,22,23,24), crops (82), natural (31,41,42,43,52,71,81,11,90,95)

# trap.data$urban #proportion of buffer 'urban' land use

# trap.data$crops #proportion of buffer 'crops' land use

# trap.data$natural #proportion of buffer 'natural' land use

## Project trap location data (used NADM83)

coordinates(trap.data) <- ~ longitude + latitude

proj4string(trap.data) <- CRS("+proj=longlat")

trap.data <- spTransform(trap.data, CRS('+init=epsg:3310'))

## Make site_code factor for use as random effect

trap.data$site_code <- as.factor(trap.data$site_code)

## Spatial set up and join of trapping and spraying data ################

## Create circle polygons ('collection area') around each included traps (5 km radius) w/ unique IDs

col.area <- lapply(1:nrow(trap.data), function(j) gBuffer(trap.data[j,], width = 5000))

for(k in 1:length(col.area)) { col.area[[k]]@polygons[[1]]@ID <- as.character(as.numeric(k)) }

## Spatial join of traps with spray polygons

# Create start/end times (days) for weekly temporal windows pre-trapping (1,2,3,4 weeks)

c.lag.start = c(0,8,15,22)

c.lag.end = c(7,14,21,28)

## Create vectors indicating days for start/end of assessing pre-spray (if trap 1-4 weeks prior to spray event)

pre.spray_start <- -seq(1, max(c.lag.end), by=7)

pre.spray_end <- -seq(7, max(c.lag.end), by=7)

## Setting up spray related columns (spatial overlap of previous sprays, product used, and prior to future sprays)

name.vect <- c(sapply(1:length(c.lag.end), function(x) paste("c.lag", x, sep="")), #% of 5km buffer around trap sprayed in indicated week prior to trapping

sapply(1:length(c.lag.end), function(x) paste("m.lag", x, sep="")), #product used in spray(s) in indicated week

sapply(1:length(c.lag.end), function(x) paste("PreSpray.", x, sep=""))) #spraying (0/1) in indicated week post trapping

trap.data <- cbind(trap.data, lapply(1:(3*length(c.lag.start)), function(x) rep(NA, nrow(trap.data))))

names(trap.data2006)[-1*(1:(ncol(trap.data2006) - length(name.vect)))] <- name.vect

## Spatial and temporal join for each trap (collection area) and each spray

sprays = list() # list to hold DiffSpray numbers

t.spray = vector() # vector to hold 'time since last spray' variable created in loops below

pre.spray = vector() # vector to hold 'time to next spray' variable created in loops below

z = ncol(trap.data) #used for assigning calculations to appropriate column below

for (i in 1:nrow(trap.data)) { # for each trapping event

for (j in 1:length(spray.spdf)) { # for each spray event polygon

t.spray[j] <- ifelse(as.character(is.na(over(col.area[[i]], spray.spdf[j,])) == FALSE)[1],

ifelse(trap.data$Year[i] == spray.spdf$Year[j],

(trap.data$Day[i]-1) - spray.spdf$Day.2014[j], 400), 400)

#if trap in spray area and same year, how long since last spray

# (trap date adjusted back 1 day to when collecting mosquitoes vs when trap was collected)

#if not in spray area or in different year, nonsense number (400)

}

pre.spray <- t.spray #save time to next spray for pre.spray assessment

t.spray <- replace(t.spray, t.spray < 0, 400) #nonesense number for negative times

# Calculate % of collection area sprayed in each week prior to trapping; gather all unique spray events in time window

for (m in 1:length(c.lag.start)) {

trap.data[i, z+m] <- ifelse(any(t.spray >= c.lag.start[m] & t.spray <= c.lag.end[m]),

gArea(gIntersection(spray.spdf[which(t.spray >= c.lag.start[m] & t.spray <= c.lag.end[m]),],

col.area[[i]])) / gArea(col.area[[i]]), 0)

# Product (aka Material) used for each timeframe if sprayed; note if at least one organophosphate/naled

# Note 'Trumpet EC' and 'Dibrom' are organophosphate/naled products used

mats <- levels(spay.spdf$Material)

trap.data[i, (z+length(c.lag.end))+m] <- ifelse(trap.data@data[i, z+m] == 0, as.character('None'),

ifelse(length(mats) == 1, as.character(mats),

ifelse("Trumpet EC" %in% mats | "Dibrom" %in% mats, as.character("Mixed.n"),

as.character("Mixed"))))

# Indicate if trap within 1-4 weeks prior to next spray (prespray)

trap.data[i,(z+2*length(c.lag.end))+m] <- ifelse(any(pre.spray <= pre.spray_start[m] & pre.spray >= pre.spray_end[m]), 1, 0)

}

}

## Set up spatial and temporal effects of aerial spraying ################

## Make PreSpray columns as factor

trap.data@data[,c(((z+2*length(c.lag.end))+1) : (z+3*length(c.lag.end)))] <-

lapply(trap.data@data[,c(((z+2*length(c.lag.end))+1) : (z+3*length(c.lag.end)))], factor)

## Spatial effect: sum of proportion sprayed over lags (average coverage per week with spray)

trap.data$c.lag1 <- replace(trap.data$c.lag1, trap.data$c.lag1 > 1, 1) #max 100%

trap.data$c.lag2 <- replace(trap.data$c.lag2, trap.data$c.lag2 > 1, 1) #max 100%

trap.data$c.lag3 <- replace(trap.data$c.lag3, trap.data$c.lag3 > 1, 1) #max 100%

trap.data$c.lag4 <- replace(trap.data$c.lag4, trap.data$c.lag4 > 1, 1) #max 100%

for(i in 1:nrow(trap.data)) {

props <- c(trap.data$c.lag1[i], trap.data$c.lag2[i], trap.data$c.lag3[i], trap.data$c.lag4[i])

trap.data$Control_space[i] <- ifelse(sum(props)==0, 0, mean(props[props > 0]))

}

trap.data$Control_space <- replace(trap.data$Control_space, trap.data$Control_space > 1, 1) #100%+ coverage -> 100%

## Temporal sequence: create factor for time (each combo of sprayed weeks as unique factor)

# left = 4 wks ago, right = 1 week ago

factor_time <- sapply(1:nrow(trap.data),

function(x) as.factor(paste(c(ifelse(trap.data$c.lag1[x] > 0, 1, 0),

ifelse(trap.data$c.lag2[x] > 0, 1, 0),

ifelse(trap.data$c.lag3[x] > 0, 1, 0),

ifelse(trap.data$c.lag4[x] > 0, 1, 0)),

collapse="")))

# Control time as factor (can use as numeric version later)

trap.data$Control_time <- factor(factor_time, levels(factor_time)[order(levels(factor_time))])

trap.data$Control_time_n <- as.numeric(trap.data$Control_time) #numeric verrsion

## Create variable indicating if at least 1 spray used during spray history in last 4 weeks

e <- length(c.lag.start) #last cumulative lag length

all.cum.mats <- lapply(1:nrow(trap.data@data), function(x) {

levels(as.factor(as.character(trap.data@data[x,(z+e+1):(z+e+e)])))})

trap.data$naled <- as.factor(sapply(all.cum.mats, function(y) ifelse("Trumpet EC" %in% y | "Dibrom" %in% y |

"Mixed.n" %in% y,

1,0)))

## Fit GAMs for each species ################

## set species of interest

sp = 'tarsalis' #options: 'pipiens', 'tarsalis'

## Fit GAMs; covariates chosen based on AIC (see Methods)

gam.mod_df <- if(sp == "tarsalis") { #tarsalis GAMs

dat_cut_fin <- trap.data[0,] #set-up

for(i in 1:length(unique(trap.data$Week))) {

dat_cut <- NULL

abund <- trap.data$tarsalis_females[trap.data$Week == unique(trap.data$Week)[i]] /

trap.data$num_trap[trap.data$Week == unique(trap.data$Week)[i]] #tarsalis per trap for that unit

cutoff <- mean(abund) + 2*sd(abund)

dat_cut <- trap.data[trap.data$Week == unique(trap.data$Week)[i],][-c(which(abund > cutoff)),]

if(!identical(dat_cut, dat_cut_fin)) {

dat_cut_fin <- rbind(dat_cut_fin, dat_cut) }

}

trap.data <- dat_cut_fin #removed top 5% of collections per week (improves fitting)

tarsalis.gam <- gam(tarsalis_females ~ offset(log(num_trap)) +

te(longitude,latitude,YearWeek, d=c(2,1), bs=c("tp","cr")) +

s(Day, bs="cc", by=urban) +

s(Day, bs="cc", by=natural) +

s(Day, bs="cc", by=crops) +

s(Temperature) +

s(av.temp_2wk) +

s(site_code, bs="re") +

PreSpray.1 + PreSpray.2 + PreSpray.3 +

te(Control_space, Control_time_n),

data = trap.data, family = nb ,method = "REML")

return(list(pipiens.gam, trap.data)) #return fitted GAM and data used in fitting

} else { #pipiens GAMs

dat_cut_fin <- trap.data[0,] #set-up

for(i in 1:length(unique(trap.data$Week))) {

dat_cut <- NULL

abund <- trap.data$pipiens_females[trap.data$Week == unique(trap.data$Week)[i]] /

trap.data$num_trap[trap.data$Week == unique(trap.data$Week)[i]] #pipiens per trap for that unit

cutoff <- mean(abund) + 2*sd(abund)

dat_cut <- trap.data[trap.data$Week == unique(trap.data$Week)[i],][-c(which(abund > cutoff)),]

if(!identical(dat_cut, dat_cut_fin)) {

dat_cut_fin <- rbind(dat_cut_fin, dat_cut) }

}

trap.data <- dat_cut_fin #removed top 5% of collections per week (improves fitting)

pipiens.gam <- gam(pipiens_females ~ offset(log(num_trap)) +

te(longitude,latitude,YearWeek, d=c(2,1), bs=c("tp","cr")) +

s(Day, bs="cc", by=urban) +

s(Day, bs="cc", by=natural) +

s(Day, bs="cc", by=crops) +

s(Temperature) +

s(av.temp_2wk) +

s(site_code, bs="re") +

PreSpray.1 + PreSpray.4 +

naled +

te(Control_space, Control_time_n),

data = trap.data, family = nb ,method = "REML")

return(list(pipiens.gam, trap.data)) #return fitted GAM and data used in fitting

}

## See Supplementary Table 1 for details on choice of splines and basis dimensions

## See R documentation for GAMs for further information on fitting, checking, and plotting

## Calculate change in abundance across spatio-temporal grid ################

## control_data is a function that returns a dataframe with the mean change in abundance for each spatio-temporal

# combination in the data along with 95% CI and indication if estimated change is significant

## model: list with GAM object and dataframe used in fitting (i.e. gam.mod_df returned above)

## step: unit used in estimation of spatial coverage (0-1); 0.01 = 1% change in spatial coverage

## naled: 0/1 indicator if to estimate change assuming at least 1 organophosphate used;

# 1 can be used only if covariate present in GAM

est.change_data <- function(model = gam.mod_df, step = 0.01, naled = 0) {

# Set-up data to use for prediction

new.dat <- expand.grid("Control_space" = seq(0,1,by=step),

"Control_time_n" = 1:length(levels(model[[2]]$Control_time))) #spatio-temporal grid

new.dat <- new.dat[-c(2:length(seq(0,1,by=step))),] #rm control_space > 0 for no spray control_time (don't need these calcs)

new.dat <- data.frame(new.dat, num_trap = 1,

longitude = unique(model[[2]]$longitude[model[[2]]$site_code == 233001]),

latitude = unique(model[[2]]$latitude[model[[2]]$site_code == 233001]),

YearDay = median(model[[2]]$YearDay),

Day = (median(model[[2]]$YearDay) - floor(median(model[[2]]$YearDay))) * 365,

YearWeek = median(model[[2]]$YearWeek),

urban = unique(model[[2]]$urban[model[[2]]$site_code == 233001]),

non.urban = unique(model[[2]]$non.urban[model[[2]]$site_code == 233001]),

natural = unique(model[[2]]$natural[model[[2]]$site_code == 233001]),

crops = unique(model[[2]]$crops[model[[2]]$site_code == 233001]),

Temperature = mean(model[[2]]$Temperature),

av.temp_2wk = mean(model[[2]]$av.temp_2wk),

site_code = 233001, naled = 0,

PreSpray.1 = 0, PreSpray.2 = 0, PreSpray.3 = 0, PreSpray.4 = 0)

# site code 233001 used most in our data, used across years, and rather central spatially

if(naled == 1) new.dat$naled[2:nrow(new.dat)] <- 1 # replace all sprayed with 1 (no spray still 0)

Xp <- predict(model[[1]], new.dat ,type="lpmatrix") #linear predictor matrix

# simulate from posterior distribution to estimate mean and variance of % change in abundance

rmvn <- function(n,mu,sig) { ## multivariante normal random deviates (need mgcv lib)

L <- mgcv::mroot(sig); m <- ncol(L);

t(mu + L%*%matrix(rnorm(m*n),m,n))

}

set.seed(04232019) #seed so same sampling each run and resulting plot

reps <- 10000 #number of samples from posterior distribution

br <- rmvn(reps, coef(model[[1]]), model[[1]]$Vc) ## 10000 replicate param. vectors,

#Note: Vc is corrected vcov for uncertanity in smooths (since using REML est method)

res <- array(0, dim=c(nrow(new.dat), 1, reps))

for (i in 1:reps) {

#for each iteration of predictions, calculate rate ratio (#/night spray/no spray) and % change

pr <- Xp %*% br[i,] ## replicate predictions

res[,,i] <- (exp(pr)/exp(pr[1])) #ratio of trap-counts/night (compared to no spray)

}

# For each cell in array (est % change), calc mean and var/SD

resp_change <- data.frame(Control_space = new.dat[,1], Control_time_n = new.dat[,2])

# Mean %change, calculated on ratio scale so symmetric around 0 and then converted to %change

resp_change$mean.chng <- sapply(1:nrow(res[,,]), function(x) mean(res[x,,])-1)

# 95% credible interval of change from posterior draws (ratio scale)

resp_change$CI_low <- sapply(1:nrow(res[,,]), function(x) quantile(res[x,,], 0.025))

resp_change$CI_up <- sapply(1:nrow(res[,,]), function(x) quantile(res[x,,], 0.975))

# Remove estimates outside the range of control_space for each sequence of sprays (control_time)

rng <- lapply(levels(model[[2]]$Control_time),

function(x) range(model[[2]]$Control_space[model[[2]]$Control_time == x])) #range of coverage for each seq of sprays

rng.ext <- lapply(rng, function(x) c(min(x) - 0.05, max(x) + 0.05)) #add little extra buffer on either side of range for plotting

rng.rnd <- lapply(rng.ext, function(x) round(replace(x, x > 1, 1), 2)) #2 digit round

rng.rnd[[1]] <- NULL #remove first entry (no spray scenario)

rng.rnd <- lapply(rng.rnd, function(x) if(-Inf %in% x) c(0,0) else x) #change -inf to inf ranges to 0,0

cov.seq <- unlist(lapply(rng.rnd, function(x) {

if(0 %in% x) { c(0,0,101) #correct assignment of in/out for control time w/o any data points

} else {

c(length(seq(0,x[1], by = 0.01))-1,

length(seq(x[1], x[2], by =0.01)),

length(seq(x[2], 1, by = 0.01))-1)

}} ))

in.cov <- which(rep(rep(c(0,1,0), time = 15), times = cov.seq) == 0) + 1 #which inside data coverage, accounting for 0 time

resp_change[in.cov, c("mean.chng","CI_low","CI_up")] <- NA #set combinations outside coverage as NA

# Number of different signs in estimated confidence interval limits to determine if crosses 0

num_sign <- sapply(1:nrow(resp_change),

function(x) length(unique(unlist(sign(resp_change[x,c("CI_low","CI_up")]-1)))))

resp_change$sig_chng <- replace(resp_change$mean.chng, num_sign == 2, NA) #NA for est with CI crossing 0

return(resp_change) #output dataframe for model with mean change, CI, and significance for spatio-temporal combinations

}
