# Supplementary material for: Spatio-temporal impacts of aerial adulticide applications on populations of West Nile virus vector mosquitoes
Source: Parasit Vectors. 2021 Feb 24;14:120. doi: 10.1186/s13071-021-04616-6 (PMC7905633; doi:10.1186/s13071-021-04616-6)
Supplement: Supplementary file 6 — Additional file 6: Table S1. Smooth functions included in the final GAMs for both Cx. tarsalis and Cx. pipiens. Table outlining the construction of smooth function used in the final models, including spline type and basis dimensions chosen. [file 13071_2021_4616_MOESM6_ESM.docx]

**Additional file 6**

**Table S1.** Smooth functions included in the final GAMs for both *Cx. tarsalis* and *Cx. pipiens.*

| **Variable(s) included** | **Biological interpretation** | **Spline chosen** | **Basis dimensions^+^** |
| --- | --- | --- | --- |
| Longitude, latitude, Week^#^ | Spatial relationships across Sacramento and Yolo counties allowed to vary on the weekly scale | Tensor product of 2D thin plate regression spline (long/lat) and cubic regression spline (time) | 125 |
| Day^†^ by ‘urban’ land use | Seasonal curve in urban areas | Cyclic cubic regression spline | 10 |
| Day^†^ by ‘crops’ land use | Seasonal curve in cultivated crop lands | Cyclic cubic regression spline | 10 |
| Day^†^ by ‘natural’ land use | Seasonal curve in non-urban non-crop areas | Cyclic cubic regression spline | 10 |
| Temperature deviation from monthly average on night of trapping (°C) | Impact of warmer/colder than normal temperatures on activity of mosquitoes on night of collection | Thin plate regression spline | 10 |
| Average temperature during previous 2 weeks (°C) | Developmental rates of mosquitoes | Thin plate regression spline | 10 |
| Spatial overlap of spray, temporal sequence of sprays | Spatio-temporal impacts of aerial spraying | Tensor product of 2 thin plate regression splines | 25 |
| Collection site | Random intercept for each collection site location | Penalized coefficient spline (identity penalty matrix) | 1,065^‡^ |

^+^ The dimension of the basis used to represent the smooth function. One degree of freedom is lost to the identifiability constraint of the smooth and the remainder sets the upper limit on the degrees of freedom associated with the smooth and was selected using the method outlined by Wood (2006).

^#^ Continuous time variable indicating the number of weeks from the start of the study period (range 1-626).

^†^ Day of the year (range 1-365).

^‡^ Number of unique collection sites.

**Reference:**

Wood SN. Generalized additive models: an introduction with R. Boca Raton, FL: Chapman & Hall/CRC; 2006.
