# Supplementary material for: Spatio-temporal impacts of aerial adulticide applications on populations of West Nile virus vector mosquitoes
Source: Parasit Vectors. 2021 Feb 24;14:120. doi: 10.1186/s13071-021-04616-6 (PMC7905633; doi:10.1186/s13071-021-04616-6)
Supplement: Supplementary file 7 — Additional file 7: Table S2. Change (%) in nightly abundance from expected for collections preceding aerial spraying. Table indicating the final model estimates for change in expected trap-counts for collections in the 1 to 4 weeks preceding an aerial spray event. [file 13071_2021_4616_MOESM7_ESM.docx]

**Additional file 7**

In order to capture dramatic deviation from ‘normal’ mosquito abundance that partially characterize high-risk periods and precipitate an aerial spray response, we considered parameters indicating the presence of an aerial spray in the following one to four weeks after a collection event in the GAMs. In other words, if a collection event precedes a spray, it would have higher abundance than expected because there is higher than average abundance and this was a factor precipitating the following spray event. These parameters captured the dramatic deviation in a way that the smooth functions describing expected abundance could not. Estimated change in abundance for retained covariates (based on reduction in AIC) in the final models for collections one to four weeks before an aerial spray are outlined in Table S2 below.

**Table S2.** Change (%) in nightly abundance from expected for collections preceding aerial spraying.

| **Species** | **PreSpray1^‡^** | **PreSpray2^‡^** | **PreSpray3^‡^** | **PreSpray4^‡^** |
| --- | --- | --- | --- | --- |
| *Cx. pipiens* | 21.9^***^ | NA | NA | 23.9^**^ |
| *Cx. tarsalis* | 13.8^*^ | 29.0^***^ | 21.0^**^ | NA |

Parameter significance: ^*^*P*<0.05; ^**^*P*<0.005; ^***^*P*<0.0001

NA: term not retained through backward selection so not present in final model.

^‡^ PreSpray#: presence of a spatially overlapping aerial spray in the indicated 1, 2, 3, or 4 weeks following a trap collection.
